# ^18^F-FDG Pet-Guided External Beam Radiotherapy in Iodine-Refractory Differentiated Thyroid Cancer: A Pilot Study

**DOI:** 10.1155/2017/9807543

**Published:** 2017-10-19

**Authors:** Eleonora Farina, Fabio Monari, Paolo Castellucci, Fabrizio Romani, Andrea Repaci, Arianna Farina, Giuseppe Zanirato Rambaldi, Giovanni Frezza, Renzo Mazzarotto, Silvia Cammelli, Luca Tagliaferri, Rosa Autorino, Francesco Deodato, Gabriella Macchia, Savino Cilla, Vincenzo Valentini, Stefano Fanti, Alessio G. Morganti

**Affiliations:** ^1^Radiation Oncology Center, Department of Experimental, Diagnostic and Specialty Medicine (DIMES), University of Bologna, S. Orsola-Malpighi Hospital, Bologna, Italy; ^2^Nuclear Medicine Unit, University of Bologna, S. Orsola-Malpighi Hospital, Bologna, Italy; ^3^Medical Physics Department, University of Bologna, S. Orsola-Malpighi Hospital, Bologna, Italy; ^4^Division of Endocrinology, University of Bologna, S. Orsola-Malpighi Hospital, Bologna, Italy; ^5^Radiology Unit, Department of Experimental, Diagnostic and Specialty Medicine (DIMES), University of Bologna, S. Orsola-Malpighi Hospital, Bologna, Italy; ^6^Radiation Oncology Unit, Bellaria Hospital, Bologna, Italy; ^7^Radiotherapy Unit, Azienda Ospedaliera Universitaria Integrata, Ospedale Civile Maggiore, Verona, Italy; ^8^Gemelli Advanced Radiation Therapy Center, Fondazione Policlinico Universitario “A. Gemelli”, Catholic University of Sacred Heart, Rome, Italy; ^9^Radiotherapy Unit, Department of Oncology, “Giovanni Paolo II” Foundation, Catholic University of Sacred Heart, Campobasso, Italy; ^10^Medical Physics Unit, “Giovanni Paolo II” Foundation, Catholic University of Sacred Heart, Campobasso, Italy

## Abstract

**Introduction:**

To evaluate the clinical response rate after a postoperative ^18^F-FDG PET/CT guided external beam radiotherapy (EBRT) in Iodine-refractory differentiated thyroid cancer.

**Material and Methods:**

Patients with thyroid cancer locally recurrent after total thyroidectomy plus metabolic radiotherapy and treated with radical EBRT were included. Inclusion criteria were detectable thyroglobulin (Tg), negative postmetabolic radiotherapy whole body scintigraphy, and no surgical indications. The pretreatment ^18^F-FDG PET/CT resulted positive in all cases (loggia, lymph nodes, and lung). EBRT was delivered with IMRT-SIB technique. A ^18^F-FDG PET/CT revaluation and Tg dosage were performed 3 months after the treatment.

**Results:**

Sixteen consecutive patients were included in this analysis (median follow-up: 6–44 months). Post-EBRT ^18^F-FDG PET/CT showed CR in 43.7%, PR in 31.2%, SD in 25.0% patients, and PD due to lung metastases in 12.5%. Overall response rate was 75.0% (CI 95%: 41.4–93.3%). Tg levels decreased in 75.0% with a median Δ of 68.0%. Two-year PFS and OS rates were 80.0% and 93.0%, respectively. Acute G3 toxicity occurred in 18.7% and late G2 toxicity in 12.5%.

**Conclusions:**

^18^F-FDG PET/CT was useful in target definition for radiotherapy planning, identifying positive areas not detected with ^131^I scintigraphy. IMRT based EBRT was feasible and our results encourage future prospective studies. This clinical trial is registered with ID: NCT03191643.

## 1. Introduction

Differentiated thyroid carcinoma (DTC) represents more than 90% of all thyroid cancers [1]. DTC usually has a good prognosis with 10-year overall survival (OS) rate of approximately 95% for papillary and 85% for follicular carcinoma [2]. Despite this excellent outcome, up to one-third of patients show tumor relapse. The great majority of recurrences occurs at locoregional level [3].

Surgical total thyroidectomy +/− central compartment and/or laterocervical lymphadenectomy, ablation with ^131^Iodine (RAI), and suppression of the Thyroid-Stimulating Hormone (TSH) are considered the standard therapy for DTC, while the role of adjuvant external beam radiotherapy (EBRT) is still discussed based on the disease low clinical aggressiveness [4–6].

After surgical treatment and ablation with ^131^Iodine, serum thyroglobulin (Tg) and anti-Tg antibodies levels should be undetectable. An increase of their values is strongly suggestive of persistent or recurrent disease [7]. To localize the site of disease recurrence (surgical thyroid bed and/or regional lymph nodes), several imaging methods are generally used: neck ultrasonography (US), CT-scan, MRI, and ^131^Iodine scintigraphy (WBS) [8–10]. Locoregional recurrence is potentially curable, especially if resectable and if it retains the ability to take up ^131^Iodine. However, there is a subgroup of recurrences with negative ^131^Iodine scintigraphy (WBS), also if performed after RAI. These neoplasms, with dedifferentiated cell features, represent approximately 2–5% of all thyroid cancers [11] and present an increased aggressiveness and a worst prognosis compared to well differentiated cancer, being responsible for a large percentage of thyroid cancer deaths [11]. This disease condition represents a “gray area” in thyroid cancer monitoring and management considering its less responsiveness to standard therapies [11]. This leads the clinicians to choose other treatments as EBRT in DTC tumors deemed unresectable. In these cases ^18^F-FDG PET can guide the physicians in therapeutic decision. In fact a positive ^18^F-FDG PET combined with high and increasing Tg serum values is an indicator of a higher histological tumor grade development and presence of locally recurrent and/or metastatic disease spread [12–14]. Its use is potentially useful not only to identify the recurrence sites, but also for target volumes definition in EBRT planning and to evaluate the disease response after therapy [15]. However, studies reporting the results of FDG PET guided EBRT in these patients are lacking.

Therefore, the aim of this study was to evaluate clinical response and outcome after EBRT in patients with locally recurrent DTC and negative ^131^I WBS.

## 2. Material and Methods

### 2.1. Study Design

This is an observational pilot study. The study was approved by the local ethics committee (Thyroid-COBRA).

### 2.2. End Points

The primary objective of the study was to evaluate the locoregional response after ^18^F-FDG PET/CT guided EBRT for patients with Iodine-refractory DTC recurrence. Secondary objectives of the study were to define progression-free survival (PFS), overall survival (OS), acute and late toxicity, and serum changes of the tumor marker (Tg value) after EBRT.

### 2.3. Eligibility

Patients with locally recurrent thyroid cancer, treated with radical EBRT from October 2011 to January 2016 after total thyroidectomy +/− central compartment and/or laterocervical lymphadenectomy, previously treated with one or more cycles of RAI and TSH suppression were included in the study. Inclusion criteria were the following: detectable serum Tg level on Levothyroxine therapy (>1 ng/ml), negative post-RAI WBS, no surgical indications, and age ≥ 18 years.

### 2.4. Radiotherapy

All patients underwent pretreatment ^18^F-FDG PET/CT in supine position with head, neck, and shoulders immobilized by a thermoplastic mask. Image fusion was used to improve the accuracy of targets contouring with coregistration of unenhanced CT images with ^18^F-FDG PET based on rigid anatomical fusion. The immobilization system was used both for treatment planning and for delivery. Planning PET/CT slices for each patients were obtained at 3 mm intervals from the vertex to the level of nearly half femur in the treatment position. Pretreatment ^18^F-FDG PET/CT resulted positive in all patients: 2 in thyroid loggia, 2 in thyroid loggia and lymph nodes, 11 only in lymph nodes, and 1 in lymph nodes and lung.

Gross tumor volume-PET (GTV-PET) was manually defined on ^18^F-FDG PET/CT images using visual interpretation as the increased FDG-uptake areas and a cut-off value of approximately 42% of the maximum intensity. Clinical target volume 1 (CTV 1) was defined as the GTV with a 5 mm expansion. Clinical target volume 2 (CTV 2) included the CTV 1, the thyroid loggia, and high risk lymph nodes (ipsilateral lymph nodes from II to VI level). Clinical target volume 3 (CTV 3) included low risk lymph nodes (contralateral lymph nodes from II to VI level). CTV 1, CTV 2, and CTV 3 were expanded into planning target volume (PTV) by adding a three-dimensional (3D) 5 mm expansion to account for setup uncertainties. In case of disease involvement of the VII neck level, patients received the radiation dose also at the upper mediastinum.

Also OARs (spinal cord, larynx, esophagus, and parotid glands) were delineated. Megavoltage EBRT was delivered with Intensity Modulated Radiation Therapy-Simultaneous Integrated Boost (IMRT-SIB) technique. IMRT plans were generated using the inverse treatment planning system (Pinnacle, Philips Healthcare, Fitchburg, WI) following the recommendation of ICRU report 83. The following dose-volume constraints were used: *D* max < 45 Gy for spinal cord and larynx, *D* mean < 34 Gy for esophagus, and *D* mean < 26 Gy for both parotid glands. Isodose distributions in three planes (axial, sagittal, and coronal) and dose-volume histograms of PTV and OARs were used to guide plans optimization.

A total dose of 66 Gy (2.2 Gy daily fraction) was prescribed to PTV 1, 60 Gy (2 Gy daily fraction) to PTV 2, and 54 Gy (1.8 Gy daily fraction) to PTV 3. All PTVs were irradiated simultaneously over 30 fractions (1 fraction/day, 5 fractions/week). Treatment was delivered with the step-and-shoot technique and 6 MV photons using an Oncor linear accelerator (Siemens Medical Solutions, Malvern, PA, United States). To ensure the correct targets positioning during treatment, we used an EPID check before EBRT start and at least once a week. The targets position was checked comparing the megavoltage portal images produced by two perpendicular square open beams at 0° (anteroposterior) and 90° (lateral) with the corresponding digitally reconstructed radiography of the same beams generated by the treatment planning system (TPS). The verification was made by comparing the position of the bony anatomy landmarks compared to the treatment field. Deviations > 3 mm in the isocenter position were corrected.

### 2.5. Follow-Up

Patients were evaluated at 1st and at 3rd month after the end of the radiotherapy and every 6 months thereafter (follow-up range: 3–44 months; median: 18.5 months). The first evaluation was clinical and included history, physical, ECOG, and toxicity assessment. The subsequent evaluations included clinical evaluation, imaging, and laboratory tests. More specifically, 3 months after IMRT completion a ^18^F-FDG PET/CT was performed to evaluate clinical response and Tg levels were assessed. The same exams were performed every 6 months thereafter.

### 2.6. Statistical Analysis

Acute and late toxicity were assessed with CTCAE v. 4.03 and EORTC-RTOG scales [16, 17], respectively. Metabolic response was scored with PERCIST criteria [18]. Local control (LC), progression-free survival (PFS), and overall survival (OS) were analyzed with Kaplan-Meier method.

## 3. Results and Discussion

### 3.1. Patients Characteristics

A total of 16 patients were consecutively treated and analyzed in our Radiation Oncology Center (M/F: 8/8; median age: 66.5 years, range: 36–81; histology: 15 papillary carcinomas and 1 follicular carcinoma; UICC stage: III-IV). Fifteen patients (93.7%) were declared unresectable while 1 patient (6.2%) refused the surgical neck lymphadenectomy. Patients and clinicopathologic characteristics and imaging and treatment data are summarized in Tables 1 and 2, respectively.

### 3.2. Response

**Figure 1:**
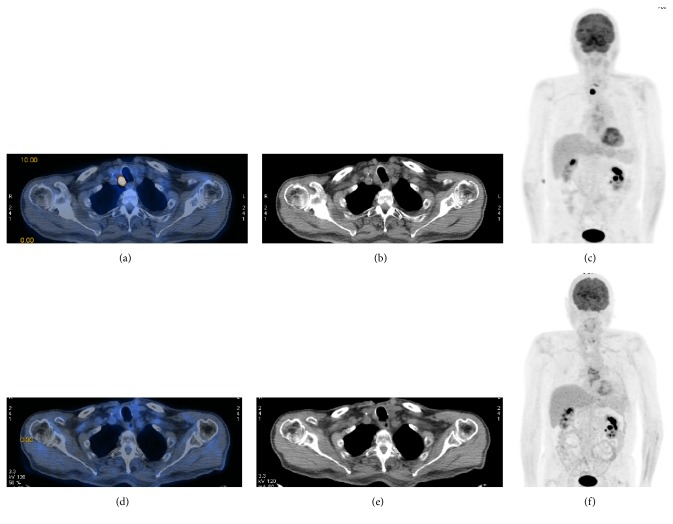
79-year-old man with metastasis ahead the trachea from classic variant PTC previously treated with surgery and RAI. (a, b, c) Pre-EBRT focal uptake (19 mm  ×  18 mm) shown with ^18^F FDG PET/CT (SUVmax 6.5), CT, and MIP-scan images, respectively. (d, e, f) Disappearance of the lesion showed with post-EBRT ^18^F FDG PET/CT, CT, and MIP-scan images, respectively.

The first ^18^F-FDG PET/CT performed after EBRT showed at locoregional level CR in 7 (43.7%), PR in 5 (31.2%), and SD in 4 (25.0%) patients. It showed also PD in 2 (12.5%) patients due to lung metastases (Table 3). Overall response rate was 75% (CI 95%: 41.4–93.3%). Clinical response (CR and PR) was 50% in local recurrences (loggia), 50% in loggia and lymph nodes, and 83.3% in nodal recurrences (*p* = .411). Metabolic pretreatment and follow-up images are shown in Figure 1. Serum Tg levels evaluated on Levothyroxine therapy at the same time resulted in the following: in 12 patients (75.0%, including one with lung metastases), Tg was decreased (median  Δ: 74.0%,  Δ  range: 14.7%–99.7% with 50% of  Δ > 50%); in 1 patient (6.2%), Tg was stable and undetectable (patient with Hashimoto disease), and in 3 patients (18.7%, including 2 with lung metastases) it was increased (median Δ: 6.4%, Δ range: 3.2%–31.7%).

### 3.3. Outcome

**Figure 2:**
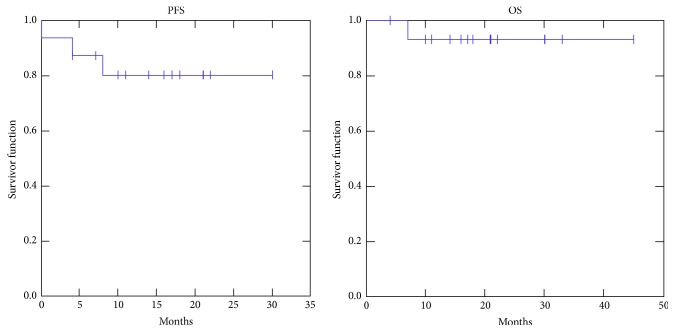
Actuarial progression-free survival (PFS) and actuarial overall survival (OS).

No patient showed local progression of the disease (LC rate 100%). Two patients (12.5%) with PR at first evaluation showed further response to therapy in terms of size and/or standard uptake value. No further distant metastases were detected during follow-up. Two-year PFS and OS were 80.0% and 93.0%, respectively (Figure 2). During follow-up, 1 patient died of myocardial infarction.

### 3.4. Toxicity

No patients presented G4 acute toxicity and the incidence of G3 acute toxicity was 18.7%. More specifically, 3 patients showed confluent and moist desquamation in the treated area. G3 skin toxicity was 0% in patients with boost delivered only on thyroid loggia, 6.2% in patients with boost delivered on the thyroid loggia and lymph nodes, and 12.5% in patients with boost only on lymph nodes (*p* = .064). In all cases, including patients with a greater treated area due to upper mediastinal irradiation, no severe skin toxicity occurred. In fact, radiotherapy was planned trying to exclude skin (minimum 5 mm) from the radiation field without bolus application. Only 1 patient (6.2%) presented acute G2 dysphagia and dysphonia with peristomal inflammation (he was the only patient with tracheostomy). G2 skin erythema was detected in 10 patients (62.5%). A complete report of acute toxicity rates is shown in Table 4.

Grade 2 late toxicity was recorded in 2 patients (12.5%) with moderate skin atrophy, telangiectasia, and moderate subcutaneous fibrosis. G1 fibrosis and G1 slight skin pigmentation were observed in 2 patients (12.5%). In particular, the 2 patients with G2 and 1 of the 2 patients with G1 late skin toxicity had previously presented a more severe acute cutaneous toxicity. Overall, a correlation between toxicity grade and site of recurrence (lymph nodes and/or loggia) was not observed.

### 3.5. Discussion

In this study, we analyzed a small series of patients treated with ^18^F-FDG PET/CT guided IMRT-SIB for locally recurrent dedifferentiated thyroid cancer. Interestingly, all patients showed a positive ^18^F-FDG PET allowing target definition for EBRT treatment planning based on this imaging technique. The treatment was well tolerated with an overall response rate of 75.0% (CI 95%: 41.4%–93.3%) and 2-year PFS and OS of 80.0% and 93.0%, respectively. The study also included 1 patient with lung metastases detected at ^18^F-FDG PET/CT simulation performed before the radiation treatment starts. For this patient, the local control was the main issue due to its importance in prognosis determination and possible cause of death for suffocation. At last follow-up, the patient showed a stable disease at both locoregional and lung level and did not receive indication to systemic therapies. Moreover, during follow-up, 2 patients presented new lung metastases and increased serum Tg levels. They were treated with stereotactic body radiation therapy (SBRT) at lung level followed by molecular targeted therapy (Sorafenib).

The study has obvious limitations mainly related to the small sample size, the retrospective design, and the short observation time. Therefore, our data on long-term treatment tolerance, tumor response, and patients outcome are only partially reliable. However, all patients were treated by the same radiation oncologists (EF, FM) and by the same nuclear medicine physicians (PC, SF) using uniform methods of treatment planning and images evaluation. Furthermore, at the best of our knowledge, this is the first report of salvage ^18^F-FDG PET/CT guided IMRT in patients with dedifferentiated locally recurrent thyroid carcinoma.

Due to the small sample size, it is difficult to compare results based on patients and tumor characteristics. We can only observe that the response rate was higher in patients with tumor in loggia and lymph nodes, that most patients (3/4) with late skin toxicity had previously shown G3 acute toxicity, and that G3 acute toxicity was higher when the boost was delivered on the lymph node area. Furthermore, we did not observe a clear correlation between tumor response and outcome, being all patients free from local tumor progression. All these differences did not reach statistical significance but they can suggest particular caution in patients with lymph nodes positivity at FDG PET because of increased risk of toxicity. Finally, the short follow-up and the small sample of patients do not allow estimating a correct evaluation of the results with prolonged follow-up.

Only few studies [5, 19–21] are available in literature about salvage EBRT in patients with locally recurrent thyroid cancer and they are summarized in Table 5. Currently, the use of EBRT is considered controversial in the salvage management of DTC due to lack of prospective randomized trials and risk of high-dose radiation treatment-related toxicity [5, 22]. We can observe that only our study included a homogeneous sample of patients with dedifferentiated recurrences, while all other studies included data on locally recurrent thyroid cancer with different characteristics. In all the studies, RAI was previously performed but the numerical estimate of patients with thyroid recurrence treated with RAI was reported only in one study (91.7%) [5]. No studies reported the results of ^131^I administrations, and consequently the rate of undifferentiated tumors is not known. Moreover, no studies reported the use of FDG PET in radiotherapy planning. Also the outcomes assessment based on different techniques and doses is not feasible because the results were reported on the entire patients cohort and there are no specific data on patients with recurrence. Therefore, the comparison among all studies remains a critical step due to heterogeneity in patient populations, methodologies, inclusion criteria, and outcome evaluation. Consequently, a quantitative comparison of salvage EBRT in patients with locally recurrent thyroid cancer cannot be performed.

## 4. Conclusion

Despite its limitations, our study suggests that ^18^F-FDG PET/CT is useful for target definition and therefore it should be used in patients with dedifferentiated thyroid carcinoma candidates for salvage EBRT. In fact it allows the identification of the relapse sites, where disease appears more aggressive and not equally identifiable with other routine instrumental tests as WBS and CT-scan. The GTV received a greater dose suitable for the radicalization of the disease. Moreover, the radiation dose delivered to the PTV included high and low risk areas. The main aim in the use of ^18^F FDG PET is to reduce the volume disease and decrease the risk of relapse in regions at risk. Furthermore, these results suggest that the technique and doses used in our experience are able to produce a high response rate and more importantly an excellent local control of the disease. These results obviously require further confirmation in prospective and larger studies with prolonged follow-up observation. Use of more advanced techniques (dose-painting IMRT-SIB based on metabolic activity) could be theoretically tested to improve the response rate.

## Figures and Tables

**Table 1:** Patients and clinicopathologic characteristics.

Case	Sex	Age at primary diagnosis	Histologic diagnosis	Stage at presentation AJCC (2010) and histological features
1	F	68	PTC (tall cell variant)	pT3 pN1b Mx R1
2	F	69	PTC (tall cell variant)	pT1b pN1b Mx R0
3	F	41	PTV (classical and follicular variant)	pT4a(m) pN1b Mx EC R2
4	F	63	PTC (classical variant)	pT3(m) pN1b Mx R0
5	M	62	PTC (classical variant)	pT4a pN1a Mx R0
6	M	40	PTC (classical variant)	pT3 pN1b Mx R0
7	F	74	PTC (classical variant)	pT4a pN1a Mx V1 R2
8	M	65	Follicular Hürthle cell carcinoma	pT4a Nx Mx R0
9	F	72	PTC (tall cell variant)	pT3 pN1a Mx R1
10	F	81	Widely invasive FTC (classical variant)	pT3 Nx Mx R1
11	M	63	PTC (classical variant)	pT1a(m) pN1b Mx EC R0
12	M	65	PTC (classical variant)	pT3 pN1b Mx V1 EC Rclose
13	M	28	PTC (classical variant)	pT3 pN1b Mx R0
14	M	78	PTC (classical variant)	pT3 pN1b Mx EC R0
15	M	70	PTC (classical variant)	pT4a(m) pN1a Mx R2
16	M	75	PTC (classical variant)	pT4a pN1b Mx EC R0

EC: extrathyroid extension; FTC: follicular thyroid carcinoma; G2: moderately differentiated; G3: poorly differentiated; m: multifocal; PTC: papillary thyroid carcinoma; R1: microscopic residual disease; R2: macroscopic residual disease; V1: blood vessel invasion.

**Table 2:** Treatments history.

Case	Surgical interventions at diagnosis	Other surgical interventions	N° RAI (total dose MBq)	Range primary diagnosis, EBRT (months)	Tg on L-T4 therapy at last recurrence (ng/ml)	Stimulated Tg at last recurrence (ng/ml)
1	TT + neck LA (central + ipsilateral)	Bilateral clavicular LA	3 (11100)	61	18.3	530.0
2	TT + neck LA (central)	Surgical neck LA (central) revision	2 (11100)	96	<0.2^*∗*^	<0.2^*∗*^
3	TT + neck LA (central + ipsilateral)	—	1 (5624)	3	1.1	41.5
4	TT + neck LA (central)	Bilateral neck LA	5 (18500)	72	5.6	294.0
5	TT + neck LA (ipsilateral)	Contralateral neck LA	3 (18620)	22	2.5	14.3
6	TT + neck LA (ipsilateral)	Ipsilateral paratracheal LA	3 (12921)	103	3.1	18.3
7	TT + neck LA (central)	—	1 (5534)	6	21.9	74.4
8	TT	Ipsilateral clavicular LA	4 (25900)	154	17.8	25.8
9	TT + neck LA (central)	—	2 (9425)	49	1.0	1.5
10	TT	—	1 (5533)	2	299.0	706.0
11	TT + neck LA (ipsilateral)	—	2 (11163)	25	3.3	19.1
12	TT + neck LA (bilateral) + partial laryngectomy	Surgical unilateral neck LA revision	3 (11290)	82	4.7	184.0
13	TT + neck LA (bilateral)	—	3 (18620)	95	114.0	388.0
14	TT + neck LA (central)	Mediastinal LA + contralateral neck LA	2 (9284)	20	3.2	27.8
15	TT + neck LA sampling (central)	—	1 (7322)	2	3.5	102.0
16	TT + neck LA (bilateral) + laryngectomy	—	1 (5714)	3	80.0	167.0

^*∗*^Presence of high AbTg levels; EBRT: external beam radiotherapy; Tg: thyroglobulin; LA: lymphadenectomy; RAI: radioiodine ablation; TT: total thyroidectomy; WBS: whole body scintigraphy.

**Table 3:** Local metabolic tumor response based on ^18^F-FDG PET (PERCIST criteria) at 1st follow-up after radiotherapy.

Case	Response
1	CR^*∗*^
2	PR
3	CR
4	SD
5	CR
6	PR
7	CR
8	SD
9	SD
10	CR
11	CR
12	SD
13	CR
14	PR
15	PR^*∗*^
16	PR

CR: complete response; LT4: Levothyroxine; RP: partial response; SD: stable disease. ^*∗*^Presence of new lung metastases.

**Table 4:** Acute toxicity after radiation treatment (maximum recorded grade).

Grade (G)	Organ tissue		
	Skin (%)	Esophagus (%)	Larynx (%)
0	0 (0.0)	8 (50.0)	10 (62.5)
1	3 (18.7)	7 (43.7)	5 (31.2)
2	10 (62.5)	1 (6.2)	1 (6.2)
3	3 (18.7)	0 (0.0)	0 (0.0)
4	0 (0.0)	0 (0.0)	0 (0.0)

**Table 5:** Clinical studies: EBRT in locoregional differentiated thyroid cancer recurrence.

Author, year	Medical center	Reference	Study design	Patients	EBRT technique (target volumes and doses)
O'Connell et al., 1994	Royal Marsden Hospital, London, UK	[19]	Retrospective	11	Bilateral neck and superior mediastinum with ^60^Co photons or 5 MV photons via AP/PA portals (60 Gy/30 daily fraction) or neck and superior mediastinum with 20 and 35 MeV electron beams, respectively (75 Gy/30 daily fraction)

Meadows et al., 2006	University of Florida Health Science Center, USA	[20]	Retrospective	20	Thyroid bed, cervical lymph nodes, upper mediastinum with photons via 3-field (anterior field of 45 Gy, opposed lateral fields with bolus to boost the final tumor dose) or thyroid bed, cervical lymph nodes, upper mediastinum with IMRT (64.9 Gy at 1.8 to 2.0 Gy/daily fraction)

Kim et al., 2010	Center for Thyroid Cancer, Research Institute and Hospital, National Cancer Center, Goyang, Korea	[21]	Retrospective	15	Limited field: recurrent tumor bed and positive nodal area via 3DCRT or IMRT (median total dose of 62.5 Gy at 1.8 to 2.5 Gy/daily fraction) or elective field: recurrent tumor bed and regional nodal areas in the cervical neck and upper mediastinum via 3DCRT or IMRT (median total dose of 50 Gy and a median dose of 62.5 Gy to boost tumor bed and positive nodal area at 1.8 to 2.5 Gy/daily fraction)

Romesser et al., 2014	Memorial Sloan Kettering Cancer Center, New York, USA	[5]	Retrospective	36	Low-risk areas to 54 Gy; high risk areas to 60 Gy; close or microscopically positive margins to 66 Gy and areas of gross disease to 70 Gy (total median dose of 66.3 Gy in a median of 33 fractions) via IMRT (majority of cases)

Current series	Bologna University, Bologna, Italy	—	Pilot	16	FDG-PET positive areas to 66 Gy, ipsilateral lymph nodes to 60 Gy, contralateral nodes to 54 Gy, in 30 fractions via IMRT-SIB technique

3DCRT: three-dimensional conformal radiotherapy; ATC: anaplastic thyroid cancer; IMRT: intensity modulated radiotherapy; yrs: years.
